# ADVANCING AFFORDABLE, ACCESSIBLE HEARING CARE FOR OLDER ADULTS: FINDINGS FROM THE HEARS RCT AND ASSOCIATED STUDIES

**DOI:** 10.1093/geroni/igae098.0430

**Published:** 2024-12-31

**Authors:** Carrie Nieman, Hae-Ra Han, Frank Lin

**Affiliations:** Chair; Co-Chair; Discussant

## Abstract

Age-related hearing loss is independently associated with adverse health outcomes, but the use of hearing aids by older adults is low and disparities exist. The incorporation of community health worker (CHW)-partnered models can reduce barriers and address disparities. The HEARS (Hearing health Equity through Accessible Research & Solutions) intervention is a hearing care intervention delivered by CHWs that incorporates a low-cost amplification device and education on age-related hearing loss. The efficacy of the HEARS intervention was assessed through a randomized controlled trial (RCT) (JAMA, 2022) that demonstrated the intervention significantly improved communication function. The RCT was the first of a CHW-delivered hearing care intervention designed for older adults that included provision of amplification. As a community-engaged trial, the trial was also one of the largest hearing-related trials in the U.S. of African American older adults and low-income older adults with hearing loss and provides a unique opportunity to advance understanding of hearing care among communities not traditionally represented within research. This symposium presents secondary analyses from the HEARS RCT that inform the continued expansion of hearing care, including among individuals with cognitive impairment and those with limited technology use. The symposium also shares findings from community-engaged hearing screening efforts (K-HEARS Screen) in partnership with Korean American ethnic churches and provides the first estimates of hearing health behaviors among older Korean Americans. Together, the symposium shares lessons in partnering with CHWs and community organizations to optimize the development and testing of interventions to advance hearing health equity across diverse communities.

